# Uneven recovery patterns of compromised health-related quality of life (EQ-5D-3 L) domains for breast Cancer survivors: a comparative study

**DOI:** 10.1186/s12955-018-0965-0

**Published:** 2018-07-20

**Authors:** Jonghan Yu, Woo-Seung Son, Sae Byeol Lee, Il Young Chung, Byung Ho Son, Sei Hyun Ahn, Min-Woo Jo, Jong Won Lee

**Affiliations:** 1 Division of Breast Surgery, Department of Surgery, Samsung Medical Center, Sungkyunkwan University School of Medicine, Seoul, 06531 South Korea; 2 Department of Preventive Medicine, University of Ulsan College of Medicine, 86 Asanbyeongwon-gil, Songpa-gu, Seoul, 138-736 South Korea; 3 Department of Surgery, University of Ulsan College of Medicine, Asan Medical Center, 86 Asanbyeongwon-gil, Songpa-gu, Seoul, 138-736 South Korea

**Keywords:** Breast cancer, Quality of life, EQ-5D-3 L, General population

## Abstract

**Background:**

Although several studies have evaluated health-related quality of life (HRQoL) in breast cancer survivors, few have compared HRQoL between breast cancer survivors and an age-matched general population in terms of improvement patterns according to time after surgery. Thus, we compared the postoperative changes in HRQoL in breast cancer survivors with those of age-matched general population groups using the EuroQoL five-dimension three-level questionnaire (EQ-5D-3 L).

**Methods:**

EQ-5D-3 L questionnaires were obtained from 686 breast cancer survivors during follow-up visits. They were divided into five groups according to time after surgery: 0–5 months, 6–11 months, 12–35 months, 36–59 months, and ≥ 60 months. Their EQ-5D-3 L data, covering five dimensions (mobility, self-care, usual activities, pain/discomfort, and anxiety/depression), were compared with those of age-matched general population groups.

**Results:**

The mean EQ-5D-3 L index of breast cancer survivors was high in group with longer time after surgery and the mean EQ-5D-3 L index of breast cancer group over 5 years after surgery was similar to that of an age-matched general population (0.919 vs 0.928, *p* = 0.305). Although there were deficits in all dimensions of breast cancer survivors, motility eventually exceeded that of general population groups and self-care and usual activities of groups over 3 years after surgery matched those of general population however, pain/discomfort and anxiety/depression of survivors over 5 years after surgery remained worse than those of the general population (*p* = 0.028, *p* < 0.001).

**Conclusions:**

Motility, self-care, and usual activities decreased in the early postoperative period for breast cancer survivors but showed recovery after 3 years. However, pain/discomfort and anxiety/depression remained poorer in these patients than in the general population for many years.

## Background

Breast cancer is the most common cancer in women worldwide [1], but its mortality has decreased due to advances in diagnostic tools and multimodal treatments. Accordingly, breast cancer patients experience various health-related quality of life (HRQoL) changes during their cancer journey, which encompasses diagnosis, treatment, long-term follow-up, and recurrence. Improved disease-free and overall survival outcomes, which are the most important achievements in breast cancer management, have led clinicians to focus intensively on the HRQoL of breast cancer survivors.

Many studies have evaluated the HRQoL of breast cancer survivors. Soon after diagnosis, there is a deficit in emotion and cognition, predominantly in younger breast cancer patients, and even many years after treatment, breast cancer survivors can have financial difficulties and show deficits in self-reported cognition, pain, and physical and social function [2–5]. Some studies reported improvements in the HRQoL of breast cancer survivors after treatment [6–12] and a long-term follow-up study showed no clinically important differences between breast cancer survivors and controls in physical functioning, fatigue, social and role functioning, emotional functioning, and symptoms [12]. Although some studies have reported on the long-term quality of life for breast cancer survivors, there have been few comparisons with the general population [6, 13, 14]. There have been several interpretations of the HRQoL of breast cancer survivors, which had various limitations according to method, follow-up period, study population, and comparison with control groups.

This investigation was conducted using the EuroQoL five-dimension three-level questionnaire (EQ-5D-3 L) as the HRQoL instrument. This tool consists of five simple questions but its validity as a HRQoL measurement approach has been confirmed for many disease areas [15–17]. In addition, the EQ-5D-3 L index can also help to later assess the efficiency of each intervention by being used as a utility score. More importantly, in Korea, the EQ-5D-3 L results of the general population have been obtained through the 5th Korea Health and Nutrition Examination Survey (KNHANES) data. Therefore, the EQ-5D-3 L is an appropriate tool for comparing the HRQoL of Korean breast cancer survivors with that of a general population.

In our current study therefore, we investigated the time-specific HRQoL after breast cancer treatment over time using the EQ-5D-3 L and compared the results with that of a large age-matched general population group. We also evaluated when the HRQoL of breast cancer survivors showed recovery and the characteristic patterns in HRQoL according to EQ-5D-3 L dimension after treatment.

## Methods

### Study design and subject recruitment

This study encompassed a cross-sectional survey. A total of 686 breast cancer patients enrolled at Asan Medical Center completed the HRQoL questionnaire during a regular follow-up visit from January 2012 to June 2012. They were disease-free at the time of the survey. Their median follow-up was 42 (1–244) months. An age-matched control group (*N* = 2744) was obtained from the 5th Korea Health and Nutrition Examination Survey (KNHANES) data for all breast cancer patients (*N* = 686) on a 4:1 basis. The KNHANES is a national, cross-sectional health examination survey and the 5th survey was conducted from 2010~ 2012. The KNHANES uses a complex, stratified, multistage probability cluster sampling to recruit a representative sample of the non-institutionalized population in Korea. It reports prevalence of various chronic conditions such as hypertension and health status using self-rated health and EQ-5D-3 L [18, 19]. This study was approved by the Institutional Review Board of Asan Medical Center. Informed consent was obtained from all participants.

### Measures

HRQoL refers to quality of life related to health conditions and perceived health conditions [20]. Generally, HRQoL is measured by a survey instrument, of which the EuroQoL EQ-5D-3 L, developed by the EuroQoL group, is the most popular. In addition, the EQ-5D-3 L was adopted by the KNHANES so national representative data were available in Korea. It consists of five dimensions: mobility, self-care, usual activities, pain/discomfort, and anxiety/depression. Each dimension is assessed on a three-point ordinal scale: no problem, some or moderate problems, and extreme problems [16]. The EQ-5D-3 L has frequently been used to measure HRQoL in the general population and in patients with different diseases such as stroke [15] and diabetes [17]. This tool also shows validity and reliability for breast cancer patients in Korea [21]. In addition, valuation sets to calculate the utility weight of the EQ-5D-3 L index score have been suggested in many countries, including England [22], the United States [23], Japan [24], and Korea [25] using the time trade-off method.

### Statistical methods

Chi-square analyses were applied to identify proportions for various characteristics by follow-up duration after surgery, to analyze differences in EQ-5D-3 L indices between two groups according to follow-up duration after surgery, and to examine problem reporting in each dimension between two groups.

## Results

### Characteristics of the study populations according to time after surgery

More than half of the current study patients had been diagnosed with early-stage breast cancer (stage I: 52.2%) and most had received breast-conserving surgery (62.2%) and radiation therapy (68.8%). Over 80% of these patients had endocrine therapy and about half had chemotherapy. For the largest proportion of patients, more than 5 years had elapsed since the surgery (32.9%). When breast cancer patients were divided according to time after surgery, the patients in the longer months after surgery were older (*p* < 0.001) and had a lower level of education (*p* = 0.014) and early stage (*p* = 0.042). In addition, a higher proportion underwent mastectomy; thus, in this group, there was a low proportion of patients who had radiation therapy. Patients in the 3 to 5 years after surgery group were the least likely to have undergone chemotherapy (39.4%) and showed the highest proportion of endocrine therapy (91.1%) (Table 1).

**Table 1:** Characteristics of patients group which was divided by time after surgery

Time after surgery, month	0–5		6–11		12–35		36–59		60-		
	N	%	n	%	n	%	n	%	n	%	*p-value*
Age at survey, year											< 0.001
Mean ± SD^a^	48.9 ± 9.7		49.1 ± 8.3		49.5 ± 8.6		49.5 ± 6.8		53.6 ± 7.9		
20–49	21	53.8	53	57.6	74	49.7	89	49.4	60	26.5	
50-	18	46.2	39	42.4	75	50.3	91	50.6	166	73.5	
Age at diagnosis, year											
Mean ± SD^a^	48.2 ± 9.7		48.1 ± 8.3		47.1 ± 8.3		45.2 ± 6.8		45.2 ± 8.1		
Level of Education											0.014
≤ middle school	10	26.3	12	13.0	20	13.5	15	8.3	40	17.7	
≤ high school	12	31.6	42	45.7	71	48.0	68	37.8	91	40.3	
≥ college	16	42.1	38	41.3	57	38.5	97	53.9	95	42.0	
Living with spouse											0.160
Yes	31	79.5	75	81.5	121	81.2	161	89.4	184	81.4	
No	8	20.5	17	18.5	28	18.8	19	10.6	42	18.6	
Stage (AJCC 7th)											0.042
I	20	51.3	42	45.7	78	52.7	111	61.7	107	47.6	
II	16	41.0	38	41.3	50	33.8	61	33.9	93	41.3	
III	3	7.7	12	13.0	20	13.5	8	4.4	25	11.1	
Type of Surgery											< 0.001
Breast conserving surgery	28	71.8	64	69.6	116	77.9	115	63.9	104	46.0	
Mastectomy	11	28.2	28	30.4	33	22.1	65	36.1	122	54.0	
Radiotherapy											< 0.001
Done	28	71.8	69	75.0	125	83.9	121	67.2	129	57.1	
Not done	11	28.2	23	25.0	24	16.1	58	32.2	96	42.5	
Chemotherapy											< 0.001
Done	23	59.0	62	67.4	87	58.4	71	39.4	143	63.3	
Not done	16	41.0	30	32.6	62	41.6	109	60.6	83	36.7	
Endocrine therapy											< 0.001
Done	29	74.4	66	71.7	112	75.2	164	91.1	200	88.5	
Not done	10	25.6	26	28.3	37	24.8	16	8.9	26	11.5	

^a,^*SD* standard deviation

### EQ-5D index changes in breast cancer survivors according to time after surgery and a comparison of EQ-5D-3 L dimensions between groups

**Fig. 1:**
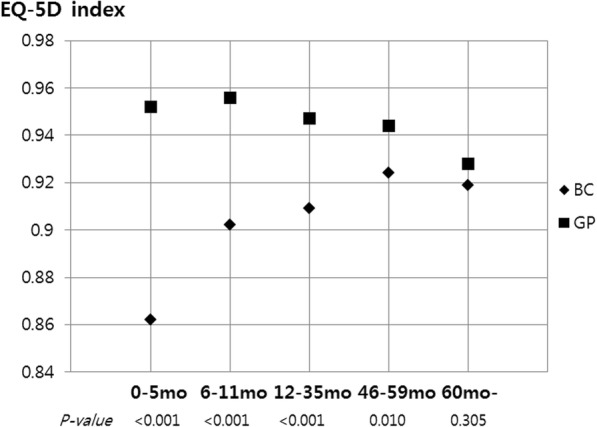
EQ-5D index changes according to time of surgery in breast cancer patients and general population (BC: Breast cancer survivors; GP:General population)

The EQ-5D indices of the breast cancer groups until 59 months after surgery were lower than those of the age-matched general population groups (Table 2). However, the EQ-5D index of the breast cancer group ≥60 months after surgery was similar to that of the age-matched general population group (0.919 vs 0.928, *p =* 0.305) (Table 2 and Fig. 1).

**Table 2:** Comparison of EQ-5D index according to time after surgery between breast cancer patient and general population

	BC^a,^ (N = 686)			Age-matched GP^b,^ (N = 2744)			
		EQ-5D index score			EQ-5D index score		
BC group according to time after surgery, month	N	Mean	SD	N	Mean	SD	*p-value*
0–5	39	0.862	0.108	156	0.952	0.078	< 0.001
6–11	92	0.902	0.081	368	0.956	0.084	< 0.001
12–35	149	0.909	0.093	596	0.947	0.110	< 0.001
36–59	180	0.924	0.075	720	0.944	0.102	0.010
60-	226	0.919	0.093	904	0.928	0.124	0.305

^a^*BC* Breast cancer survivors, ^b^*GP* General population

A comparison of the proportion of individuals indicating a problem in the breast cancer and age-matched general population groups according to the five dimensions of the EQ-5D revealed that the breast cancer group within 6 months after surgery was more likely to indicate a problem than the age-matched general population group in all dimensions. In particular, over half of the patients in the immediate postoperative period expressed a problem in the pain/discomfort (64.1%) and anxiety/depression (53.8%) dimensions. However, the proportion of breast cancer group survivors indicating a problem lowered in all aspects as time passed after surgery.

**Fig. 2:**
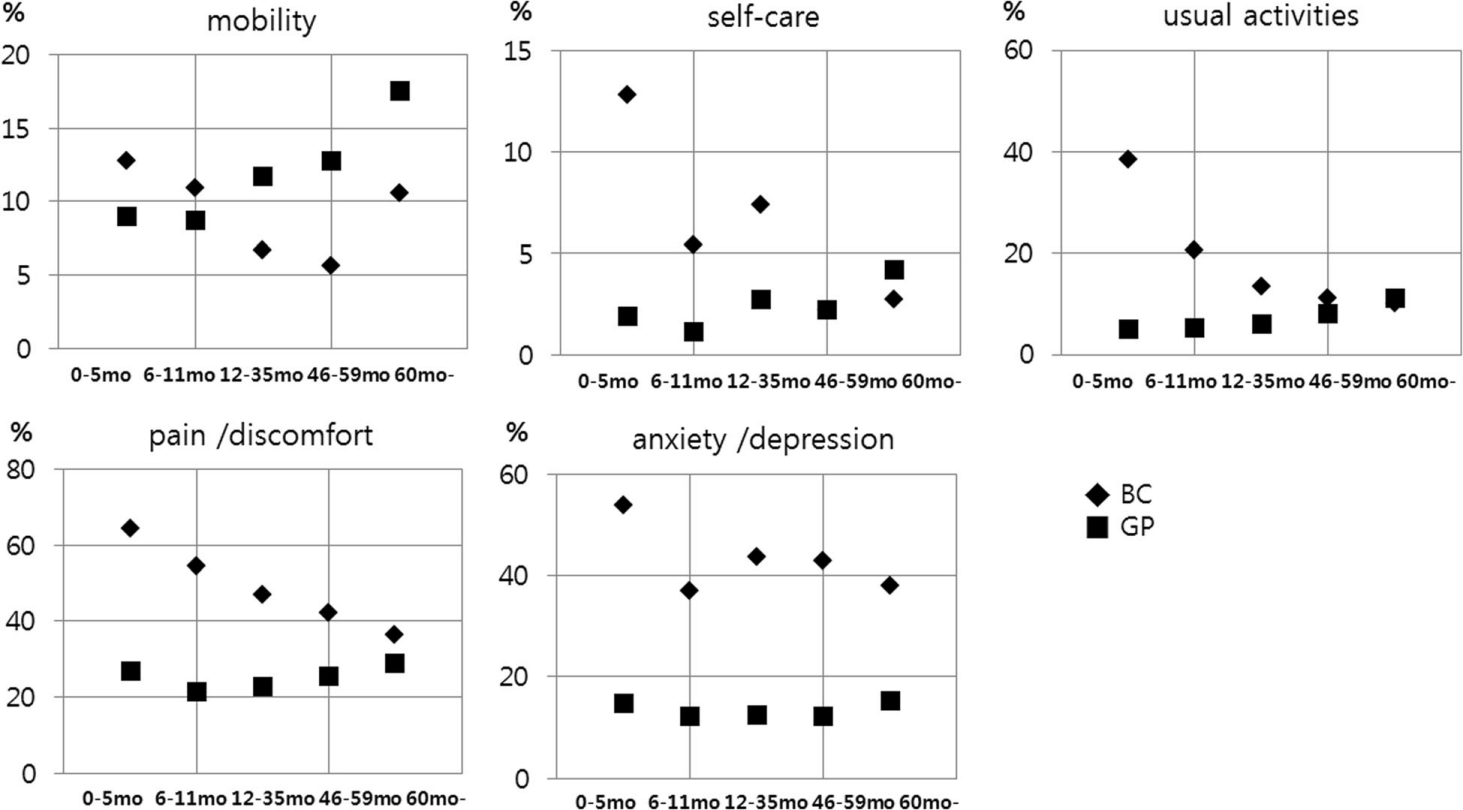
Change in the proportion of individuals indicating a problem in each EQ-5D category according to time after surgery in breast cancer patients and general population (BC: Breast cancer survivors; GP:General population)

Interestingly, the breast cancer group at 36–59 months and ≥ 60 months after surgery was significantly less likely to indicate a problem in the mobility dimension than the age-matched general population groups (5.6% vs 12.8%, *p* = 0.006; 10.6% vs 17.5%, *p* = 0.012). In the self-care and usual activities dimensions, breast cancer group over 3 years after surgery and age-matched general population showed similar proportions in a problem category, but a problem was indicated by a high proportion in all breast cancer group for the pain/discomfort and anxiety/depression dimensions (Table 3, Fig. 2).

**Table 3:** Comparison of proportion of “problem” status in each EQ-5D category according to time after surgery between breast cancer patients and general population

BC according to time after surgery, month		BC^a^ 0–5	Age-matched GP^b^	*p-value*	BC 6–11	Age-matched GP	*p-value*	BC 12–35	Age-matched GP	*p-value*	BC 36–59	Age-matched GP	*p-value*	BC 60-	Age-matched GP	*p-value*
mobility	problem(%)	12.8	9.0	0.469	10.9	8.7	0.517	6.7	11.7	0.076	5.6	12.8	0.006	10.6	17.5	0.012
	no problem(%)	87.2	91.0		89.1	91.3		93.3	88.3		94.4	87.2		89.4	82.5	
self-care	problem(%)	12.8	1.9	0.002	5.4	1.1	0.007	7.4	2.7	0.006	2.2	2.2	1.000	2.7	4.2	0.282
	no problem(%)	87.2	98.1		94.6	98.9		92.6	97.3		97.8	97.8		97.3	95.8	
usual activities	problem(%)	38.5	5.1	< 0.001	20.7	5.2	< 0.001	13.4	6.0	0.002	11.1	8.1	0.192	10.2	11.1	0.702
	no problem(%)	61.5	94.9		79.3	94.8		86.6	94.0		88.9	91.9		89.8	88.9	
pain /discomfort	problem(%)	64.1	26.9	< 0.001	54.3	21.2	< 0.001	47.0	22.7	< 0.001	42.2	25.4	< 0.001	36.3	28.8	0.028
	no problem(%)	35.9	73.1		45.7	78.8		53.0	77.3		57.8	74.6		63.7	71.2	
anxiety /depression	problem(%)	53.8	14.7	< 0.001	37.0	12.2	< 0.001	43.6	12.4	< 0.001	42.8	12.1	< 0.001	38.1	15.3	< 0.001
	no problem(%)	46.2	85.3		63.0	87.8		56.4	87.6		57.2	87.9		61.9	84.7	

^a^*BC* Breast cancer survivors; ^b^*GP* General population

## Discussion

The EQ-5D index of the breast cancer group in our current study at ≥60 months after surgery was similar to that of an age-matched general population group. The dimension of mobility was significantly better in breast cancer groups over 36 months after surgery than in age-matched general population groups, whereas the self-care and usual activities dimensions were similar. However, the categories of pain/discomfort and anxiety/depression were poorer than in the matched general population groups, with the deficits sustained over 5 years after surgery.

Unlike our results, Stover et al. showed the decrements of survivors within 6 months of diagnosis generally improved in QOL (pain, social functioning, physical functioning, etc) after 12 months in 65 years and older American breast cancer patients [26]. However, considering that mean age of all Korean patients groups in this study was under 55 years, the recovery time and patterns of deficit after cancer treatment may be different depending on race and age.

There was a significant difference in the EQ-5D index among the groups according to time after surgery. As the time after surgery was longer, the EQ-5D index was higher and the HRQoL level of the breast cancer group over 5 years after surgery reached that of the age-matched general population group. In Table 2, the EQ-5D index at each domain was similar between breast cancer group and age-matched general population. And we can consider minimally important difference as the effect size in EQ-5D index results. Minimally important difference (MID) utility was various on diseases or other conditions but it is generally reported from 0.028–0.124 [27, 28]. This result showed under 0.020 of MID between breast cancer over 3 years after surgery and age-matched general population. It was that there was no significant difference of the HRQoL level between two groups.

However, the mean age at the survey of the group with a long follow-up time (≥5 years) after surgery was older than that of the other breast cancer groups and older people generally have a lower EQ-5D index than young people. Therefore, their index was expected to be lower than that of the other follow-up period groups. Against our expectations, the older group with a long follow-up time after surgery had a higher EQ-5D index than other younger groups with a short follow-up time after surgery. In addition, the group with a long follow-up time had more patients who underwent mastectomy and chemotherapy than the short-term follow-up groups. Thus, we expected patients in the long follow-up time group to show a low EQ-5D index. Mastectomy is associated with lower psychological well-being and overall quality of life than breast-conserving therapy even many years after treatment [6] and because chemotherapy can lead to the development of acute symptoms such as vomiting, fatigue, and alopecia, it has a negative impact on HRQoL [29]. Other systematic reviews showed that past chemotherapy was one of the strongest significant predictors of a poor quality of life [14, 30]. However, survivors with a long follow-up time had a high score that was similar to that of the age-matched general population group. This mismatched result may be because the time and adaptation after treatment seemed to have a greater effect on the HRQoL of patients than age, diagnosis, and treatment, which generally affect HRQoL. Therefore, psychologic adaptation during the time after surgery may be crucial to explaining the improving HRQoL of cancer patients.

One interesting aspect of this study was that the proportion of patients indicating a problem in the dimension of mobility in the breast cancer group at 36–59 months and ≥ 60 months after surgery was significantly lower than in the age-matched general population groups. In other words, physical improvement and adaptation seemed to influence the gradual increase in HRQoL in breast cancer survivors. Increased physical activity after treatment has previously been positively associated with psychosocial well-being and HRQoL in breast cancer survivors [31]. In addition, exercise has beneficial effects on the HRQoL of cancer survivors, as assessed at various follow-up times [32].

Despite of the high EQ-5D index, assessment of the proportion of individuals indicating a problem showed different outcomes according to EQ-5D dimension. In the mobility dimension, the breast cancer group over 3 years after surgery were less likely to indicate a problem than the age-matched general population group, as discussed above, and breast cancer group after 3 years and age-matched general population showed the similar proportion of individuals with a problem in the usual activity and self-care dimensions. In terms of pain/discomfort and depression/anxiety, although the proportion of individuals indicating a problem slowly decreased, the restriction persisted, even over 5 years after surgery. That is, breast cancer survivors experienced showed the recovery of HRQoL related to physical health up to that of the general population. However, they were likely to still have a low HRQoL level in pain and psychological aspects. Previous work also showed that deficits in such aspects as emotion or cognition persisted for many years in women with breast cancer [2] and some studies reported that breast cancer survivors experienced relative declines in HRQoL, even many years after diagnosis [3, 4]. This result suggested that soon after an operation, a physical intervention such as mobility rehabilitation and regular exercise is needed to improve the HRQoL of breast cancer patients but intervention for and psychological support and pain management should consistently be provided to patients for a long time after surgery.

This study has the limitations of a cross-sectional study. This was a cross-sectional survey at one healthcare system vs. national survey for general population and we could not identify results based on longitudinal data in the same patient cohort. And there are many factors such as gender, age, education level, income that can affect the quality of life. Because all of the patients were female, we did not consider gender in this study. Age was used as a factor. However, other factors were not taken into consideration. This point is also a limitation of this study.

However, this assessment was conducted in large-scale breast cancer patient groups and an age-matched Korean general population as a healthy control group, only enrolled patients with a long postoperative follow-up time, and provided the HRQoL of breast cancer survivors according to various times from surgery, comparing the data with those of age-matched general population groups. Therefore, these outcomes revealed the change of HRQoL according to EQ-5D dimension and which level of the general population the patients’ HRQoL reached. Moreover, this study provided the EQ-5D index, which could be used as a utility score to assess the efficiency of intervention programs provided to breast cancer survivors in future work. This would help us to design the prospective tailored HRQoL intervention programs that support breast cancer survivors according to time from operation and provide basic information to combine new technologies and conventional study of HRQoL by using diverse instruments such as mobile and wearable devices to monitor the HRQoL of breast cancer survivors.

## Conclusions

This study was a cross-sectional survey at one healthcare system vs. national survey for general population.

Although breast cancer survivors had a low EQ-5D index at an early stage after surgery, the index changed over time, eventually index of breast cancer survivors over 5 years after surgery showed similar to the age-matched general population group. However, the HRQoL level of the breast cancer group even over 5 years after surgery in the pain and psychological aspect was still lower than that of the age-matched general population group, even though the HRQoL of the physical area was similar to that of the general population. Therefore, to improve the HRQoL of breast cancer patients, tailored intervention programs that depend on time after surgery and dimension should be designed. This study also provides data for assessing the efficiency of such tailored HRQoL intervention programs. Prospective large-scale cohort studies of breast cancer survivors are needed to provide definite evidence to define the factor(s) affecting HRQoL according to time and to investigate the benefits of tailored interventions.

## Abbreviations

EQ-5D-3 L: EuroQoL five-dimension three-level questionnaire
HRQoL: Health-related quality of life
KNHANES: Korea Health and Nutrition Examination Survey
